# Cell Autonomous and Non-Autonomous Functions of IKKβ and NF-κB during the Pathogenesis of Gastrointestinal Tumors

**DOI:** 10.3390/cancers3022214

**Published:** 2011-04-28

**Authors:** Hsin-Yu Fang, Florian R. Greten

**Affiliations:** Institute of Molecular Immunology, Klinikum rechts der Isar, Technical University Munich, Ismaninger Str. 22, Munich 81675, Germany

**Keywords:** NF-κB, inflammation, gastrointestinal cancer

## Abstract

Genetic studies describing a link between cancer and inflammation have increased recently. Activation of the transcription factor nuclear factor-κB (NF-κB) and its effector pathways has been proposed to be the missing link between these two processes. NF-κB is persistently activated in several types of tumors. However, NF-κB has a distinct role in cancer cells and in inflammatory cells. While in tumor cells NF-κB controls cell survival, in inflammatory cells NF-κB activates genes that encode pro-inflammatory cytokines which further act in a paracrine manner within the tumor microenvironment to contribute to tumorigenesis. Inactivation of NF-κB can also reduce chemoresistance and radioresistance of cancer cells. Therefore, specific NF-κB inhibition in combination with cytotoxic drugs and/or irradiation represents a very promising strategy for cancer therapy.

## 1. Introducton

NF-κB was first described as a B-cell-specific transcription factor that binds the κB site in the immunoglobulin (Ig) κ light chain enhancer region. However, it is now known that NF-κB has multiple critical roles in the regulation of immune responses and influences gene expression events that impact cell survival, differentiation, and proliferation. The family of NF-κB proteins consists of five members, p50 (p105), p52 (p100), p65 (RelA), c-Rel, and RelB, encoded by the genes *NF-κB1*, *NF-κB2*, *RELA*, *REL*, and *RELB*, respectively. All these molecules share an N-terminal Rel homology domain (RHD) that is responsible for DNA binding and homo- and hetero-dimerization [1,2].

NF-κB dimers bind to κB sites within the promoters/enhancers of target genes and regulate transcription through the recruitment of co-activators and co-repressors. The transcription activation domain (TAD) necessary for the positive regulation of gene expression is present only in p65, c-Rel, and RelB. Because p50 and p52 lack TADs, they can repress transcription unless associated with a TAD-containing NF-κB family member or other proteins capable of co-activator recruitment [1] Constitutive binding of p50 or p52 homodimers to κB sites on NF-κB-responsive promoters may thus act to check NF-κB transactivation until displaced by transcriptionally competent NF-κB dimers.

## 2. Different Pathways Leading to NF-κB Activation

**Figure 1.**
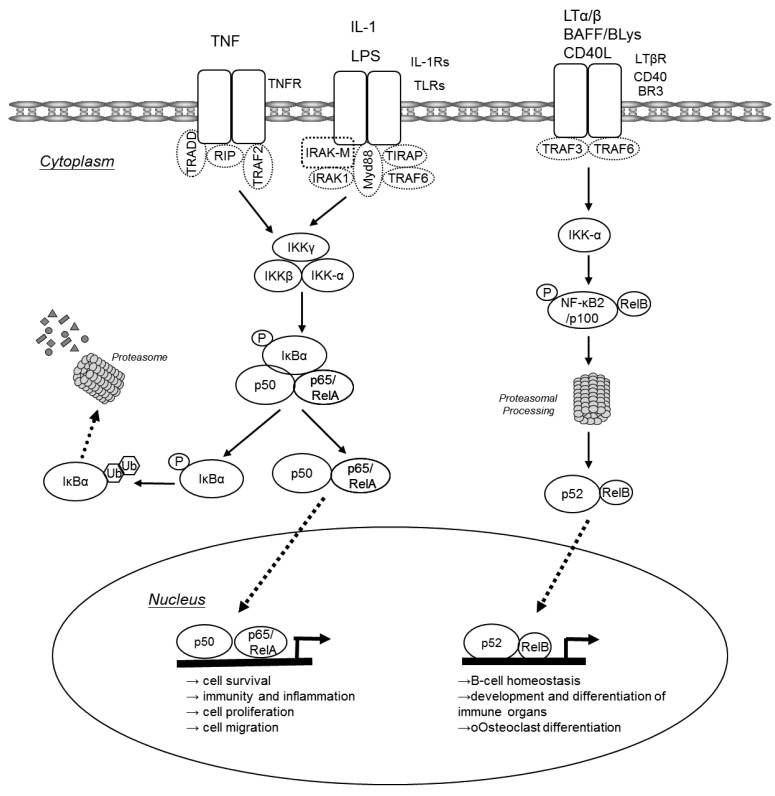
The two NF-κB activation pathways. The classical NF-κB pathway (left-hand side) results in translocation of primarily p50/p65 dimers in an IKKβ/IKKγ dependent manner. The alternative pathway for NF-κB (right-hand side) results in nuclear translocation of p52-RelB dimers and is strictly dependent on IKKα homodimers.

In unchallenged cells NF-κB dimers are bound to inhibitory IκB proteins retaining them in the cytoplasm. Two major pathways lead to translocation of NF-κB dimers from the cytoplasm to the nucleus: the canonical pathway and the alternative pathway (Figure 1). The canonical NF-κB pathway is triggered by pro-inflammatory cytokines or pathogen-associated molecular patterns (PAMPs) that engage different receptors belonging to the TNF receptor (TNFR), IL-1 receptor (IL-1R) or Toll-like receptor (TLR) superfamilies. Via various adaptor molecules signaling impinges on the IκB kinase (IKK) complex, which acts as the central regulator of NF-κB activation. The IKK complex is comprised of two catalytical subunits IKKα (IKK1 or CHUK) and IKKβ (IKK2) as well as the regulatory subunit IKKγ (NEMO). In case of the canonical pathway, the activated IKK complex phosphorylates IκBs in an IKKβ/IKKγ dependent manner. These phosphorylated IκBs are now subject to poly-ubiquitination and consecutive proteasomal degradation, thus liberating p50/p65 heterodimers that can now translocate to the nucleus to initiate transcription of genes that are involved in cell survival, immunity and inflammation, cell proliferation and cell migration. Furthermore, activated p50/65 dimers activate genes encoding chemokines, cytokine and adhesion molecules that are important for innate immune response to invading microorganisms and are required for migration of inflammatory and phagocytic cells to tissues where NF-κB has been activated in response to infection or injury [2,3]. NF-κB dimers also assure the re-synthesis of IκB proteins that serve as negative feedback inhibitors. In contrast, the alternative NF-κB pathway is triggered by different TNF superfamily members such as BAFF, lymphotoxin-β or CD40L. These agonists induce processing of p100 to p52 via activation of IKKα, resulting in the nuclear translocation of p52-RelB. The alternative pathway has been suggested to play a central role in development and maintenance of secondary lymphoid organs. The canonical NF-κB pathway may also be indirectly linked to the alternative NF-κB pathway and may influence the amplitude and duration of its activation [2,4-6]. Increased p100 processing also contributes to the malignant phenotype of certain T- and B-cell lymphomas.

## 3. NF-κB in Tumorigenesis

NF-κB is persistently activated in several types of hematological and solid malignancies [7,8]. While only recently loss of function mutations in *NFKBIA* have been described in glioblastoma patients [9], most sporadically occurring solid tumors do not harbor any mutations in genes encoding key members of this pathway. In contrast, in various lymphoid malignancies chromosomal translocations, amplifications, deletions and mutations effecting genes coding for NF-κB and IκB proteins can result in constitutive NF-κB activity [10]. In the absence of activating mutations, the canonical NF-*κ*B pathway is commonly activated by an autocrine or paracrine cytokine secretion in solid tumors. Persistent NF-κB signaling within the tumor microenvironment confers enhanced tumor cell survival and proliferation as well as stimulation of invasive growth [11]. Moreover, chronic inflammation that has been shown to increase the risk of various malignancies [12] is invariably associated with the activation of NF-κB and its effector pathways. Therefore, NF-κB has been suggested to be the missing link between these two processes. In the following sections we will review evidence demonstrating the various NF-κB controlled mechanisms with a special focus on the pathogenesis of different gastrointestinal tumors.

## 4. NF-κB in Gastrointestinal Cancer

### 4.1. Colon Cancer

The first functional genetic *in vivo* evidence of NF-κB having a direct and indirect role in tumorigenesis came from murine model of colitis-associated carcinogenesis (CAC) when IKKβ was specifically deleted in intestinal epithelial cells (IECs)—Which are the cells that undergo malignant progression—Or in myeloid cells [13]. Deletion of IKKβ in IEC or in myeloid cells reduces tumor incidence by about 75% and 50% respectively. However, NF-κB affects tumorigenesis differently in IEC and myeloid cells. IEC-specific IKKβ deletion results in enhanced p53/JNK-independent apoptosis during early tumor promotion which leads to the elimination of initiated enterocytes, thus decreasing tumor incidence, yet leaving tumor size unaffected. In addition, during acute colitis NF-κB is also responsible for the recruitment of macrophages that secrete cytoprotective pro-proliferative cytokines such as IL-11 and IL-22 [14]. In contrast, in myeloid cells IKKβ/NF-κB control production of paracrine signaling molecules or angiogenesis factors such as IL-1β, IL-6, TNF-α and COX-2 that promote proliferation of tumor cells [13]. This is further supported by findings in *Cyld*-deficient mice. Cylindromatosis (CYLD) is a deubiquitinating enzyme supposedly downregulating NF-κB activity by the proteolysis of K63-linked ubiquitin from proximal NF-κB signaling constituents such as TNF receptor-associated factor 2 (TRAF2), TRAF6, and IKKγ. In line with being a negative regulator of NF-κB, *Cyld*-deficient mice show elevated CAC incidence presumably caused by increased production of NF-κB dependent pro-inflammatory cytokines in macrophages [15]. Thus, NF-κB has a dual and very cell type specific function in CAC development.

### 4.2. Gastric Cancer

Gastric adenocarcinoma is one of most common cancers in the world and is strongly linked to chronic inflammation. Gastric cancer is thought to be induced to a large extent by the *Helicobacter pylori* (*H. pylori*) [16]. *H. pylori* can activate NF-κB leading to IL-8 secretion in human gastric epithelial cell lines [17]. Moreover, nuclear NF-κB was seen in gastric epithelial cells (GECs) of patients with *H. pylori* infection and correlated with neutrophil influx [17,18]. Functional support for a role of NF-κB in *Helicobacter* infection and cancer development came from a mouse study. *Helicobacter felis* infection in mice with a deletion of IKKβ in GECs resulted in the accumulation of myeloid cells that promoted progression of gastric neoplasia. However, loss of IKKβ in myeloid cells inhibited development of gastric atrophy by decreasing TNF-α, IL1-α, IL-1β, MMP-9 and COX-2 expression [19]. Presumably, IL-1 is one of the most important cytokines for gastric cancer development. Stomach-specific IL-1β overexpression leads to accumulation of myeloid-derived suppressor cells (MDSCs) and the development gastric neoplasia through the IL-1R/ NF-κB pathway [20]. Furthermore, in a model of *N*-methyl-*N*-nitrosourea (MNU)-induced gastric cancer, IKKβ regulates GEC apoptosis via transcriptional control of cIAP2, A20 and Bcl-2 as well as IL-1α secretion. GEC-restricted deletion of IKKβ increased apoptosis and decreased IL-1α, which correlated with the attenuation of tumor incidence [21]. Thus, similarly, to its function in CAC, NF-κB in GEC controls cell survival and recruitment of myeloid cells, while in macrophages it is mainly regulating pro-inflammatory cytokine production, which can stimulate proliferation in a paracrine manner.

### 4.3. Liver Cancer

Hepatocellular carcinoma (HCC) is closely associated with chronic inflammatory liver diseases, such as those caused by viral infection, alcohol consumption, or hepatic metabolic disorders. Several mouse models have provided evidence that activation of NF-κB in different cell types plays an important role in the pathogenesis of HCC as well. Extensive NF-κB activation caused by TNFα was found in Mdr2 knockout mice, which spontaneously develop cholestatic hepatitis followed by HCC [22]. NF-κB inhibition by the inducible transgenic expression of a non-degradable IκBa mutant in hepatocytes did not affect tumor initiation but blocked tumor promotion and progression [22].

However, in a model of chemically induced liver carcinogenesis, NF-κB plays a more complex role and mice lacking IKKβ in hepatocytes exhibited even an increased tumor load when challenged with diethyl nitrosamine (DEN). This correlated with enhanced reactive oxygen species (ROS) production, prolonged c-Jun N-terminal kinase (JNK) activation and augmented compensatory proliferation of surviving hepatocytes [23]. ROS production can cause DNA mutations in epithelial cells that contribute to genetic instability [24]. These epithelial cells are often rapidly dividing due to continual tissue repair mechanisms that occur at chronic inflammatory sites and any genetic mutations caused by ROS can rapidly divide. The anti-oxidant butylated hydroxyanisole (BHA) can prevent ROS accumulation thus preserving MAPK phosphatases (MKPs) activity, which ultimately inhibits sustained JNK activation [25]. Accordingly, BHA consumption decreased hepatocarcinogenesis in mice lacking of IKKβ in hepatocytes suggesting that DEN-induced liver carcinogenesis in these mice were mostly likely via its ability to inhibit ROS accumulation [23]. The importance of JNK in this context was later confirmed using JNK-deficient mice, that were less susceptible to DEN-induced HCC development and which reversed the susceptibility to DEN-induced HCC caused by hepatocyte-restricted deletion of IKKβ [26]. In line with these findings, Beraza *et al.* showed that loss of IKKγ alone in hepatocytes triggered chronic inflammation and spontaneous hepatocellular carcinoma development, which could be prevented by BHA administration [27].

**Figure 2.**
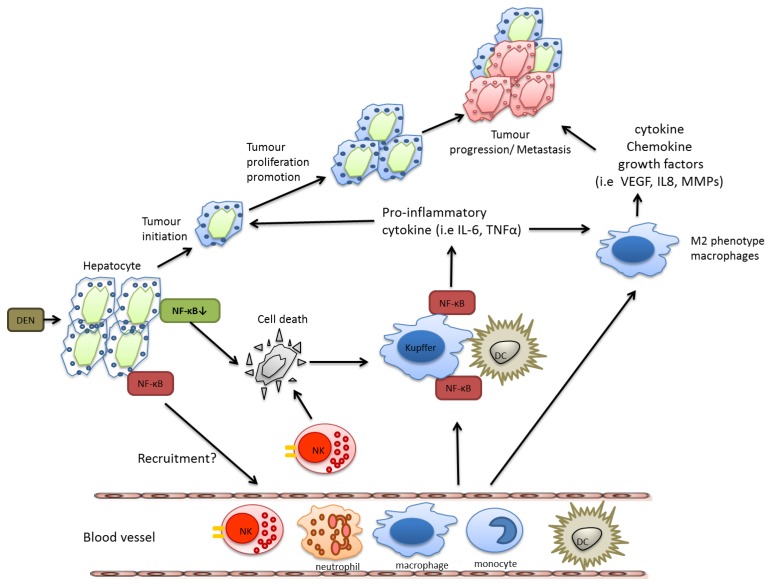
In IKKβ-deficient hepatocytes, necrotic cell death of DEN-exposed hepatocytes is augmented leading to the release of pro-inflammatory factors triggering IKK-β and NF-κB signaling in adjacent myeloid cells, which release pro-inflammatory cytokines and hepatomitogens that ultimately promote growth of DEN-initiated hepatocytes.

Nevertheless, as described above for colon and gastric cancer, during HCC development NF-κB activation in resident myeloid cells is pro-tumorigenic. In DEN-challenged mice, Kupffer cells secrete TNF-α and IL-6 and have the capacity to activate hepatic stellate cells to release hepatomitogens [23]. In summary, also during HCC development IKKβ and NF-κB confer distinct functions in specific cell types, which are summarized in Figure 2.

## 5. Does the IKKβ Dependent NF-κB Signaling Pathway Represent a Therapeutic Target?

NF-κB is constitutively activated in a large variety of human tumors. Recent mouse genetic studies have helped to unravel the diverse molecular mechanisms of this transcription factor in different cell types and have provided strong evidence that pharmacological inhibition of this signaling cascade may be an attractive novel strategy for the treatment of inflammation associated gastrointestinal tumors but also for a large variety of other tumors [10]. Although under certain circumstances, a cell type specific inhibition of NF-κB may for example increase the risk of HCC development, it is unlikely that pharmacological IKKβ inhibitors may have the same effects considering that such compounds would not target only hepatocytes, but would block NF-κB activation in inflammatory cells as well. Whether such specific IKKβ inhibitors however will be successful as single agents needs to be evaluated. Nevertheless, due to the potential of NF-κB inhibitors to increase sensitivity to chemotherapeutic drugs and irradiation based on studies performed in xenograft models [28], combination therapies may represent an excellent strategy for the therapy of advanced cancer stages and may allow the administration of lower doses of cytotoxic drugs, which could also decrease the unwanted side effects of such substances. Importantly, due to the great impact on cells of the B and T cell lineage [29] long-term IKKβ inhibition can cause loss of adaptive immunity. Moreover, NF-κB's recently described role in negatively regulating IL-1β processing that is also causally involved in the pathogenesis of granulocytosis [30,31], could also enhance susceptibility to develop septic shock upon bacterial infections in patients that use IKKβ inhibitors over extended time periods or at high doses. Thus, the usage of specific IKKβ inhibitors as cancer preventive agents may be restricted to cancer therapy either alone or in combination with cytotoxic drugs. In any case the effects on the immune system have to be monitored very closely.

## 6. Conclusions

Considering the promising data obtained from various tumor studies in genetically engineered mice, it will be essential to explore whether such novel IKKβ inhibitory molecules that are currently being developed will prove to be efficacious anti-carcinogenic compounds for the treatment of human cancer patients. Exciting times are lying ahead of us when we will determine whether the preclinical results will hold their promise once translated into the clinics.
